# Label-free photoacoustic computed tomography of visually evoked responses in the primary visual cortex and four subcortical retinorecipient nuclei of anesthetized mice

**DOI:** 10.1117/1.NPh.11.3.035005

**Published:** 2024-07-30

**Authors:** Kai-Wei Chang, Xueding Wang, Kwoon Y. Wong, Guan Xu

**Affiliations:** a University of Michigan, Department of Biomedical Engineering, Ann Arbor, Michigan, United States; b University of Michigan, Department of Radiology, Ann Arbor, Michigan, United States; c University of Michigan, Department of Ophthalmology and Visual Sciences, Ann Arbor, Michigan, United States; d University of Michigan, Department of Molecular, Cellular and Developmental Biology, Ann Arbor, Michigan, United States

**Keywords:** photoacoustic computed tomography, retinal photostimulation, hemodynamic response, photoreceptor degeneration, melanopsin

## Abstract

**Significance:**

Many techniques exist for screening retinal phenotypes in mouse models in vision research, but significant challenges remain for efficiently probing higher visual centers of the brain. Photoacoustic computed tomography (PACT), with optical sensitivity to hemodynamic response (HR) in brain and ultrasound resolution, provides unique advantages in comprehensively assessing higher visual function in the mouse brain.

**Aim:**

We aim to examine the reliability of PACT in the functional phenotyping of mouse models for vision research.

**Approach:**

A PACT-ultrasound (US) parallel imaging system was established with a one-dimensional (1D) US transducer array and a tunable laser. Imaging was performed at three coronal planes of the brain, covering the primary visual cortex and the four subcortical nuclei, including the superior colliculus, the dorsal lateral geniculate nucleus, the suprachiasmatic nucleus, and the olivary pretectal nucleus. The visual-evoked HR was isolated from background signals using an impulse-based data processing protocol. *rd1* mice with rod/cone degeneration, melanopsin-knockout (mel-KO) mice with photoreceptive ganglion cells that lack intrinsic photosensitivity, and wild-type mice as controls were imaged. The quantitative characteristics of the visual-evoked HR were compared.

**Results:**

Quantitative analysis of the HRs shows significant differences among the three mouse strains: (1) *rd1* mice showed both smaller and slower responses compared with wild type (n=10,10, p<0.01) and (2) mel-KO mice had lower amplitude but not significantly delayed photoresponses than wild-type mice (n=10,10, p<0.01). These results agree with the known visual deficits of the mouse strains.

**Conclusions:**

PACT demonstrated sufficient sensitivity to detecting post-retinal functional deficits.

## 1 Introduction

Mouse models are popular in vision research due to their phylogenetic similarity to humans and the availability of versatile genetic tools.^1^ Researchers analyze the phenotypes of various mouse models of human diseases to learn how these diseases might impact visual functions and to assess therapeutic efficacy. Many techniques for screening retinal phenotypes in these mice exist,^2^ but significant challenges for efficiently probing higher visual centers of the brain remain. Although microelectrode recording, two-photon imaging, and immunostaining of activity-dependent gene expression provide cellular resolution, these time-inefficient methods are impractical for comprehensive phenotypic screening of the visual system. Thus, many researchers have turned to simple behavioral assays such as the pupillary light reflex and optokinetic reflexes, but these subcortically driven responses do not assess image-forming vision, and rapid functional assays remain unavailable for most visual nuclei.

A promising approach for comprehensively phenotyping the mouse visual system is the imaging of visual-evoked hemodynamic responses (HRs), which are highly correlated to neural activities.^3^ Importantly, these volumetric imaging methods can theoretically analyze most if not all of the >20 brain visual areas simultaneously, thereby dramatically boosting the efficiency of post-retinal functional phenotyping of mouse models. Functional magnetic resonance imaging (fMRI), based on the blood-oxygen-level-dependent (BOLD) effect, has sufficient spatial and temporal resolution to resolve hemodynamics in the superior colliculus (SC), dorsal lateral geniculate nucleus (LGd), and primary visual cortex (V1) in mice;^4^ however, its high cost and enclosed system configuration restrict applications in vision research with small animals. Functional near-infrared spectroscopy (fNIRS) possesses a contrast mechanism comparable to fMRI, but with higher sensitivity to hemodynamics.^5^ fNIRS exhibits excellent mobility and temporal resolution in imaging human brain activities, substantially expanding the scope of brain research in the past decade.^6^ With spatial resolution limited by photon diffusion to a few millimeters, fNIRS, however, cannot resolve most visual nuclei in the mouse brain.^5^

Photoacoustic computed tomography (PACT) breaks the photon diffusion limit by combining the contrast mechanism of fNIRS and the spatial resolution of ultrasound imaging. PACT systems can image up to hundreds of frames per second^7^ with a resolution of about 100  μm at a depth of 1 cm.^8^ PACT has revealed resting-state functional connectivity in multiple cortical areas^9^^,^^10^ and captured cortical responses to intense electrical stimulations with^11^ or without optical contrast agents.^12^ PACT has successfully recorded visual responses in V1 cortex of mice^13^ and rats^14^^,^^15^ and more recently in the mouse SC.^16^ In PACT-US parallel imaging, the PACT images are naturally co-registered with the US images as the transducer array acquires both simultaneously, thereby allowing the HR to be easily superimposed onto the brain anatomies revealed by US imaging.

Our recent pilot study confirmed the feasibility of PACT-US parallel imaging to capture visually evoked HRs in V1 and SC of wild-type mice.^16^ The present study used a linear array with a mechanical translation to cover major visual regions of high interest to researchers, including the V1, SC, LGd, suprachiasmatic nucleus (SCN), and olivary pretectal nucleus (OPN). In addition, we established a data processing method for isolating the visual-evoked responses from background signals. Rod/cone-degenerate (*rd1*) and melanopsin-knockout (mel-KO) mice were used to investigate the performance of our system.

## 2 Materials and Methods

### 2.1 Mouse Preparation

All animal procedures were approved by the Institutional Animal Care and Use Committee at the University of Michigan. Three mouse strains were used: homozygous *rd1* mice, mel-KO mice,^17^ and C57BL/6 wild-type mice. All animals were aged 2 to 3 months, and both sexes were studied to avoid any sex-related bias. Animals were kept in a 12-h light/dark cycle, with PACT conducted during the light phase. Under anesthesia by isoflurane (4%) and analgesia by carprofen (5  mg/kg s.c.) plus topical bupivacaine (2  mg/kg), the scalp of the animal was removed to minimize optical and acoustic attenuation, and the skull was slightly thinned using a micro-drill to reduce acoustic attenuation.^18^^,^^19^ The mouse was then returned to its housing cage to recover from the surgery and to dark-adapt overnight. Just before PACT imaging, the mouse was anesthetized by 1% isoflurane in conjunction with acepromazine (5  mg/kg i.p.)^20^ and secured to a multi-axis translation stage with its cortex surface aligned perpendicular to the imaging plane. Imaging and photostimulation were performed as described in Secs. 2.2 and 2.3. At the end of the imaging session, the mouse was euthanized using cervical dislocation while still under anesthesia, in accordance with the guidelines of the Unit for Laboratory Animal Medicine at the University of Michigan.

### 2.2 Imaging System

**Fig. 1:**
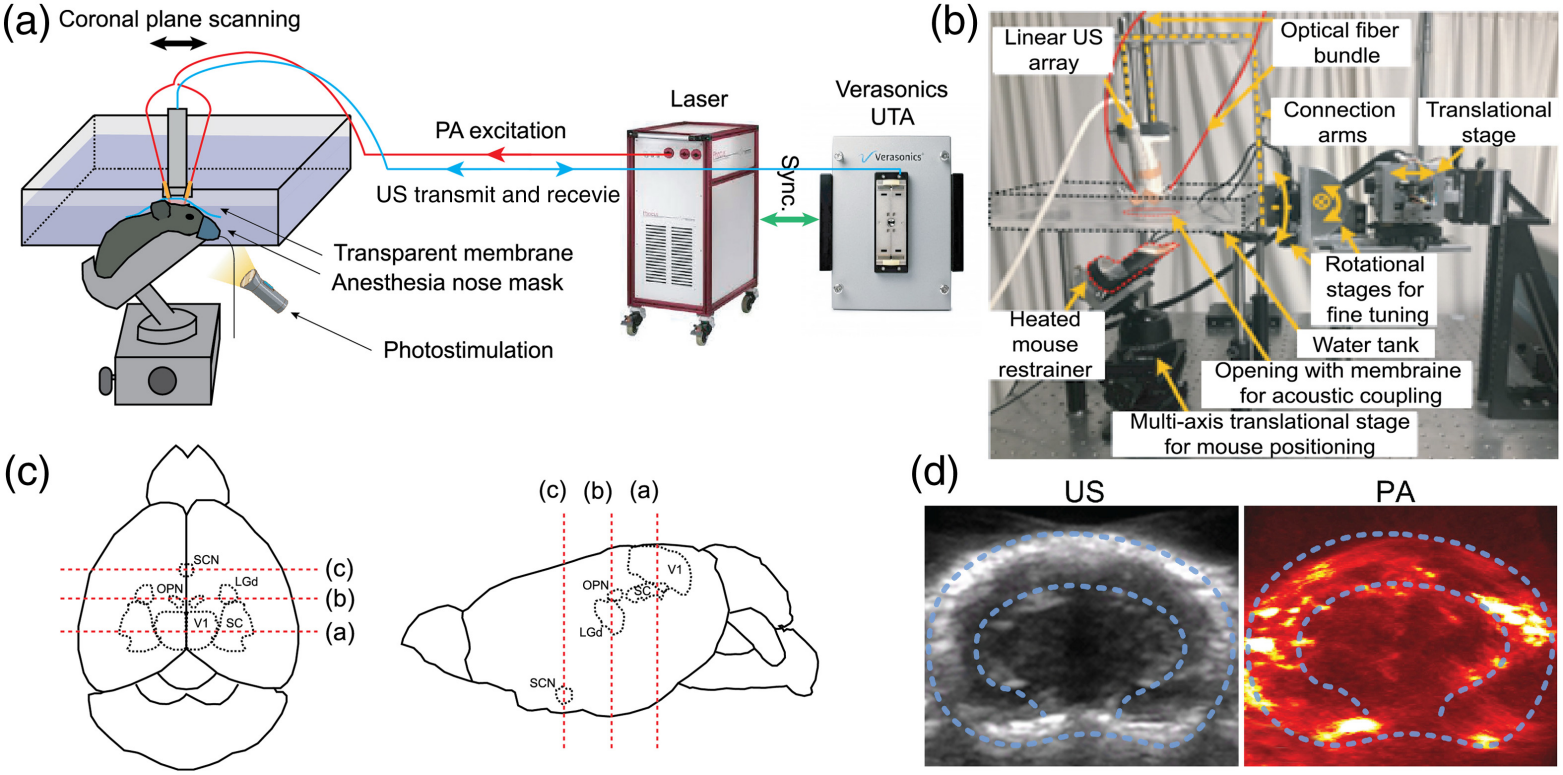
System overview. (a) A schematic diagram of the PACT system. (b) A photograph of the system and the translational stage for coronal plane imaging. (c) The positions of the targeted brain nuclei: V1, primary visual cortex; SC, superior colliculus; LGd, dorsal lateral geniculate nucleus; OPN, olivary pretectal nucleus; and SCN, suprachiasmatic nucleus. The three imaging planes are indicated with red dashed lines. (d) Representative US and PACT images acquired by the system. The dashed contours illustrate the contours of the skull and inner brain.

Figure 1(a) shows a schematic of our PACT system for real-time brain imaging. This system was used in our previous study^16^ and is briefly described here. A neodymium-doped yttrium aluminum garnet laser-pumped optical parametric oscillator (Phocus MOBILE, OPOTEK Inc., Carlsbad, California, United States) was used as the excitation source, with a pulse duration of 5 to 7 ns, a pulse repetition rate of 10 Hz, and a pulse energy variation below 5%. A 797-nm laser beam was delivered through a bifurcated, multimode fiber bundle with fiber tips attached on both sides of a linear 256-element ultrasonic array (GE L8-18i) with a central frequency of 10 MHz. A 797-nm laser wavelength was chosen because (1) mouse retinal photoreceptors are insensitive to near-infrared wavelengths,^21^^,^^22^ so the imaging illumination would not interfere with the visual stimulation and (2) at 797 nm, the oxygenated and deoxygenated hemoglobin have the same absorption coefficients, thereby facilitating the calculation of total hemoglobin levels. The US array integrated with fiber optics was positioned 1 cm away from the skull surface. The maximum optical fluence at the skull surface was $∼20\;\mathrm{mJ}/{\mathrm{cm}}^{2}$, below the American National Standards Institute safety limit of $31\;\mathrm{mJ}/{\mathrm{cm}}^{2}$ at 797 nm.^23^ The US transducer array was mechanically translated among three imaging planes containing the brain regions of interest, at a frame rate of ∼3.3  Hz for each imaging plane. The PA signals were digitalized and sampled at 40 MHz with a Vantage 256 ultrasound research system (Verasonics, Redmond, Washington, United States), which has an axial resolution of 150  μm and a lateral resolution of 150  μm at a 2-cm imaging depth, roughly corresponding to the distance between the US array and the bottom of the mouse brain.

### 2.3 Retinal Photostimulation

The visual stimulus was broadband white light generated by a fiber-optic halogen illuminator (HL150-B, AmScope, Old Bridge, New Jersey, United States) flickering at 1 Hz and positioned about 5 cm from each eye, producing an irradiance of $∼16\;\mathrm{mW}/{\mathrm{cm}}^{2}$ at the cornea. Before photostimulation, the dark-adapted mouse was kept in constant darkness for 10 min for baseline measurement. Then, both eyes were exposed to the flickering stimulus simultaneously for 10 s, followed by another 10 min in darkness, during which PACT imaging continued to monitor hemodynamic recovery from the photoresponse.

### 2.4 Image Reconstruction and Signal Processing

The PACT images were reconstructed using a delay-and-sum beamforming algorithm from the acquired signals. The temporal trace of each pixel was extracted from the PACT video as 200 consecutive frames covering a 1-min period, with 67 frames (i.e., 20 s) immediately before stimulation, 33 frames during stimulation, and 100 frames immediately after stimulation. Each 200-frame trace was first detrended by subtracting the linear fitting line of the pre-stimulation temporal trace to remove the systematic shift from the detected signal. Then, it was normalized by the root mean square of the signal strength of the original pre-stimulation temporal trace before subtraction, resulting in the baseline-subtracted and normalized PA signals (ΔPA/PA). A spatial moving average of 3×3  pixels and a temporal forward-moving average of eight frames (2.7 s) were also applied to filter out the noise from random fluctuations. After signal processing, the vessel features of the PACT images were removed, while the temporal traces of the PA amplitude changes remained.

### 2.5 Isolation of the HR

We established a data processing protocol to isolate the HR from the background noise in PA signals, building upon established techniques in fMRI and fNIRS.

A bandpass filter ranging from 0.01 to 1.25 Hz is applied to the frequency domain spectra of the PA signals. This step is crucial for reducing repetitive noises stemming from physiological activities such as respiration and heartbeats, as well as low-frequency baseline signals.^24^ We then implement a Kalman filter to eliminate autocorrelated measurement noises.^24^

**Fig. 2:**
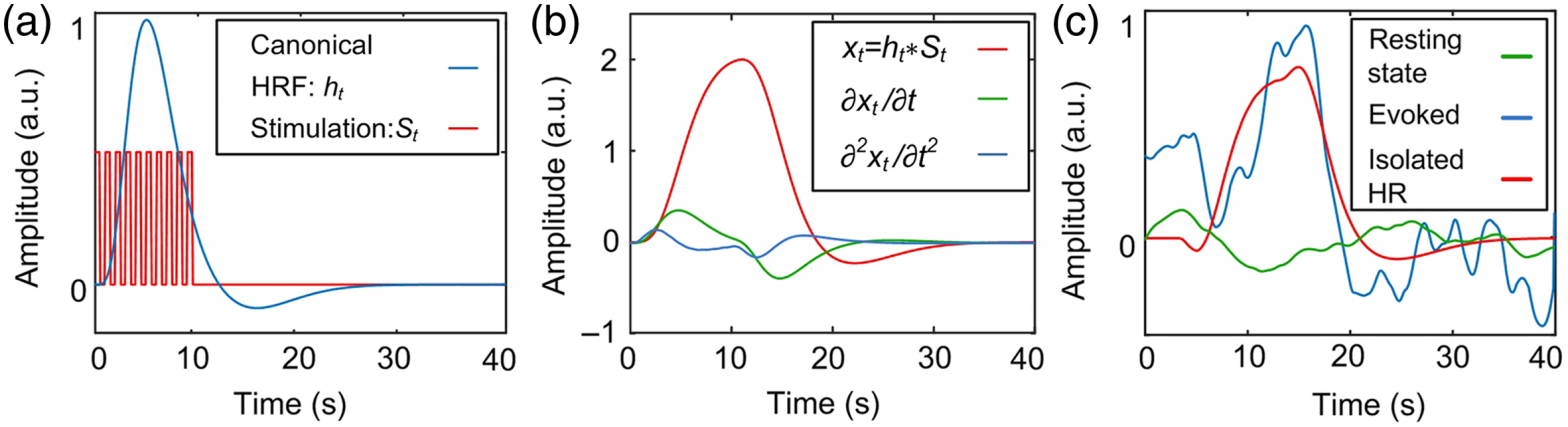
HR isolation from PA signals. (a) Canonical function and visual stimulation sequence. (b) A basis set derived from panel (a). (c) Resting state and visually evoked temporal trace in PA image and the isolated HR.

To model the visual-evoked HR, we use linear basis function modeling based on a fixed canonical-shaped HR,^25^^–^^27^ a model commonly utilized in fMRI^28^ and fNIRS.^29^^,^^30^ This function models the impulse HR to neuronal activation, as shown in Fig. 2(a). The impulse HR is convolved with our photostimulation sequence, forming the basis function, as shown in Fig. 2(b).

The HR is then modeled as a linear combination of a basis set, following standard methods.^31^^–^^36^ The model is expressed as

yt=β^0+β^1xt+β^2∂xt∂t+β^3∂2xt∂2t+εt,

where β^0 is a constant bias, β^1xt+β^2∂xt∂t+β^3∂2xt∂2t represents the second-order Taylor expansion of the basis function, and $\varepsilon_{t}$ is a noise term. Considering that some mouse strains with visual impairments exhibit delayed responses, we fit the temporal traces at each pixel in the PA image to a series of basis sets with 1-s shifts by adjusting βi^. The basis set yielding the least fitting error and the lowest p-value in the linear fit is selected as the isolated HR, as shown in the red trace in Fig. 2(c).

## 3 Results

### 3.1 Functional Maps of Visual-Evoked Hemodynamic Changes

**Fig. 3:**
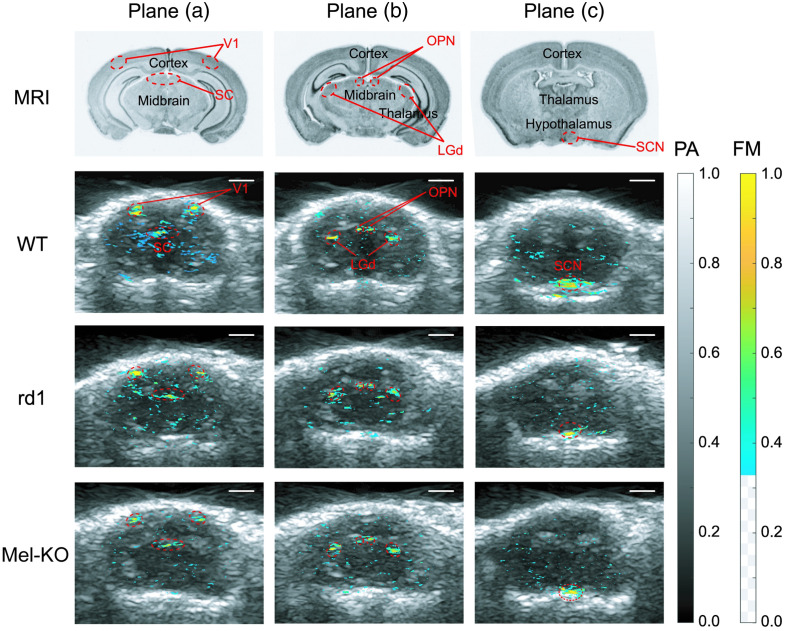
Visual regions of interest and the FMs acquired by the PACT-US system. The three columns of images correspond to the three imaging planes in this study. Row 1: MRI images from the Allen Brain Atlas at the imaging planes. The visual regions of interest are marked with red dashed ovals. Rows 2 to 4 are representative visual-evoked FMs derived from PA images overlaid on the US images in wild-type, *rd1*, and mel-KO mice, respectively. The contours of the targeted nuclei are marked with red dashed ovals. Scale bars: 2 mm.

Figure 3 row 1 shows MRI images from the Allen Brain Atlas (Allen Institute for Brain Sciences, Seattle, Washington, United States). By comparing with anatomies in those images, the five visual areas of interest were located in the US images. To generate functional maps (FMs, color-coded pixels in Fig. 3 rows 2 to 4), we calculated the maximum relative variations of the averaged signal amplitude at each pixel of the temporal PA images during and after stimulation (20 to 60 s) compared to the averaged signal amplitude before stimulation (0 to 20 s). The color-coded pixels were co-registered with the US images to confirm that the areas with the most robust visually evoked hemodynamic changes matched the locations of the five brain regions of interest (Fig. 3).

### 3.2 Temporal Traces of Visually Evoked Hemodynamic Changes

**Fig. 4:**
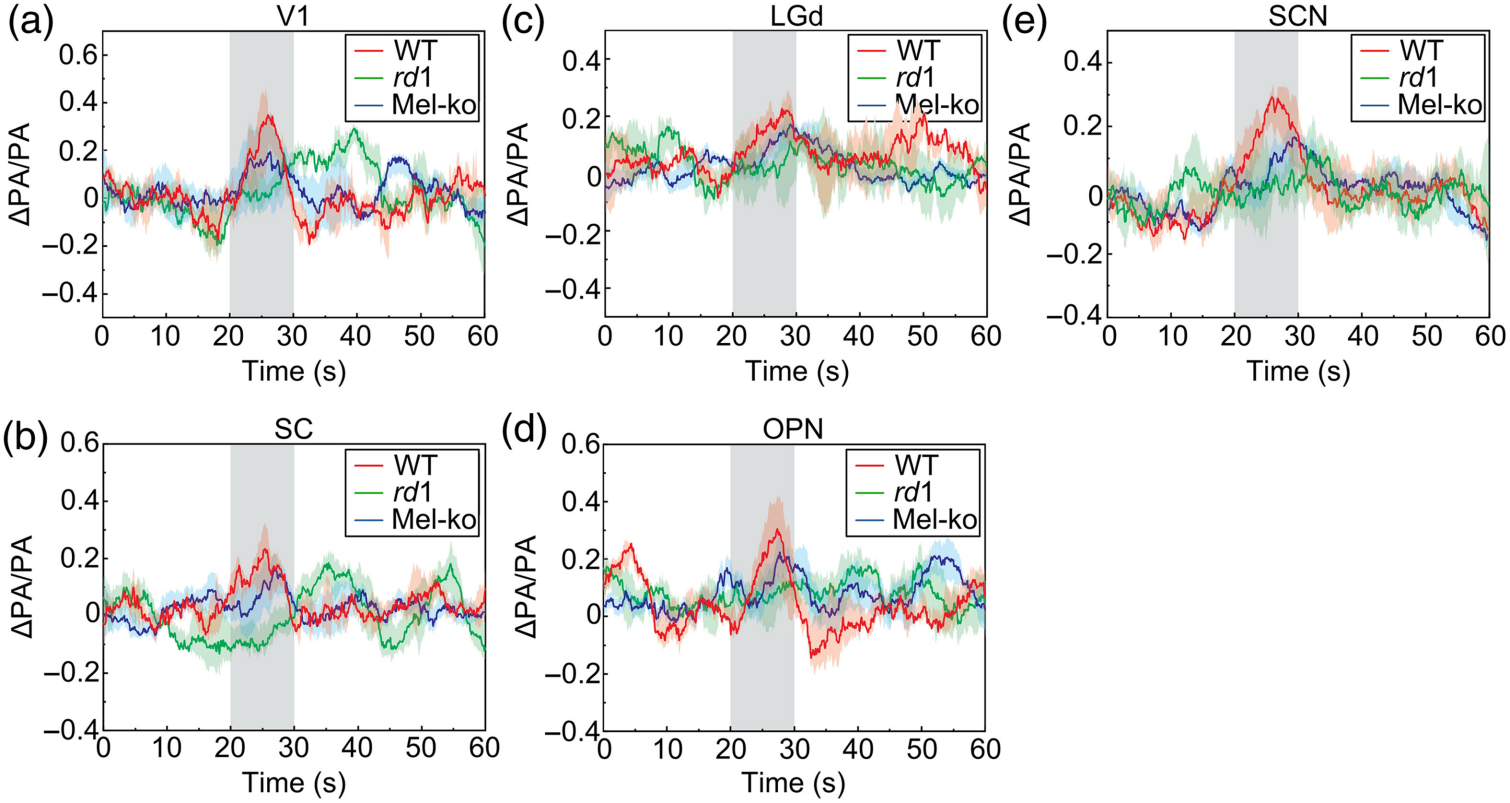
Visually evoked hemodynamic changes detected in V1 (a), SC (b), LGd (c), OPN (d), and SCN (e) of the three mouse strains. Shaded areas at 20 to 30 s represent the period of stimulation. In each panel, each trace plots the mean PA signals (ΔPA/PA) measured over the 60-s protocol for one of the mouse strains: 10 wild-type mice (red), 10 *rd1* mice (green), and 10 mel-KO mice (blue). To generate each trace, the single pixels with the highest visual-evoked changes were first identified in the corresponding visual region of each mouse, with the mean pre-stimulation PA signals normalized to zero, and the 10 mice’s temporal traces were averaged. The standard deviations of the PA signals are shown as the upper and lower bounds of the traces.

**Fig. 5:**
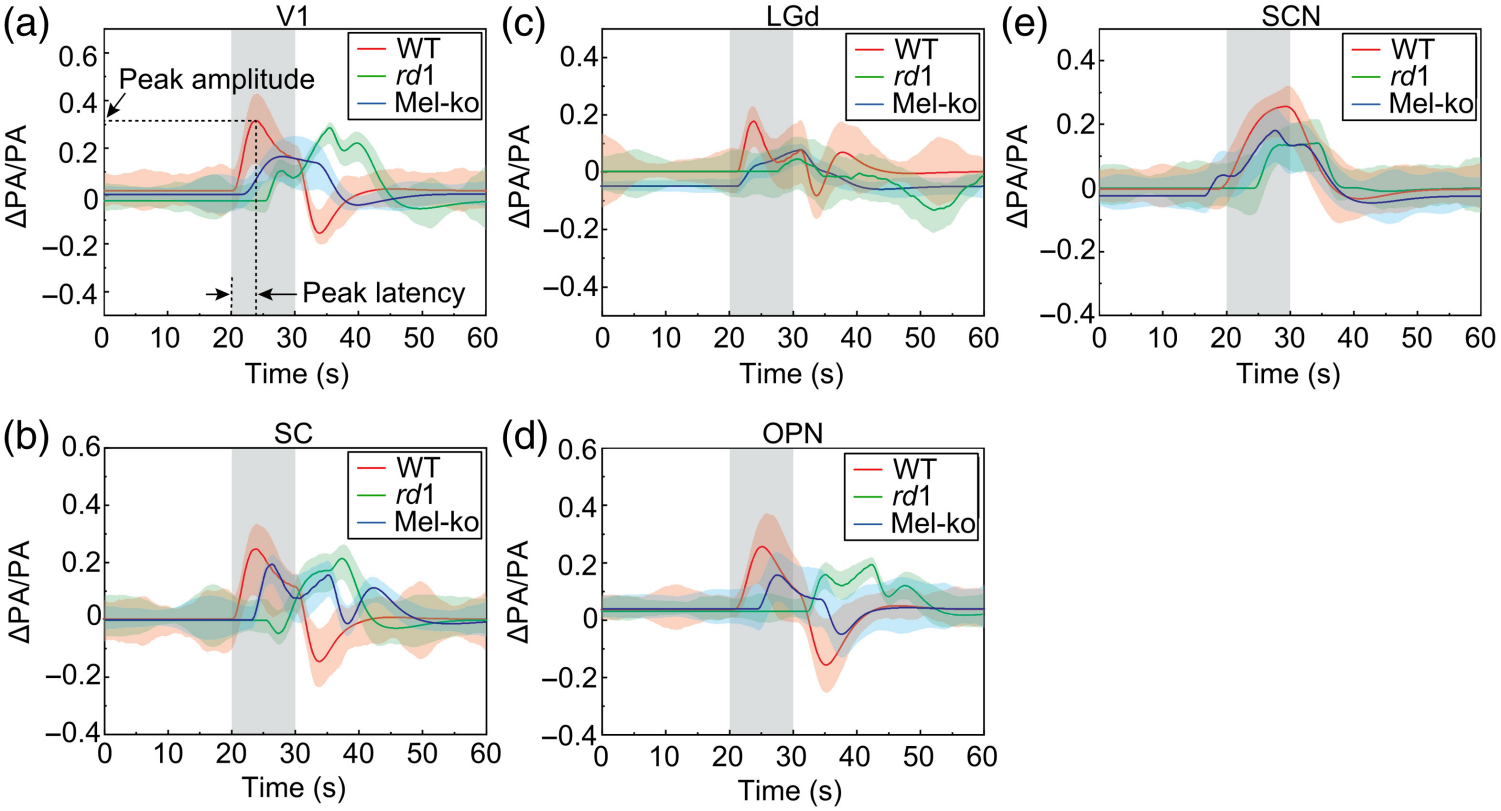
Isolated visual-evoked hemodynamic changes in V1 (a), SC (b), LGd (c), OPN (d), and SCN (e) of the three mouse strains. The traces are population-averaged temporal traces of the background-subtracted and normalized PA signals (ΔPA/PA) for single pixels with the highest functional changes located in the targeted brain areas of 10 wild-type mice (red), 10 *rd1* mice (green), and 10 mel-KO mice (blue). The standard deviations of the temporal traces are shown as the upper and lower bounds of the traces. Shaded areas represent the period of stimulation. Two properties of the visual-evoked responses were quantified as illustrated in panel (a), and the results are presented in Fig. 6.

Five males and five females of each mouse strain were studied, and all five targeted brain regions were examined in each mouse. The average temporal traces are shown in Fig. 4, and the average isolated HRs are in Fig. 5. To quantify visual deficits in *rd1* and mel-KO mice, we measured the peak amplitude and latency of the isolated HRs, as illustrated in Fig. 5(a): the peak amplitude was calculated by measuring the difference between the peak PA signal intensity and the mean pre-stimulation intensity, and the peak latency was measured as the time at which the signal reached the maximum intensity after the onset of photostimulation.

**Fig. 6:**
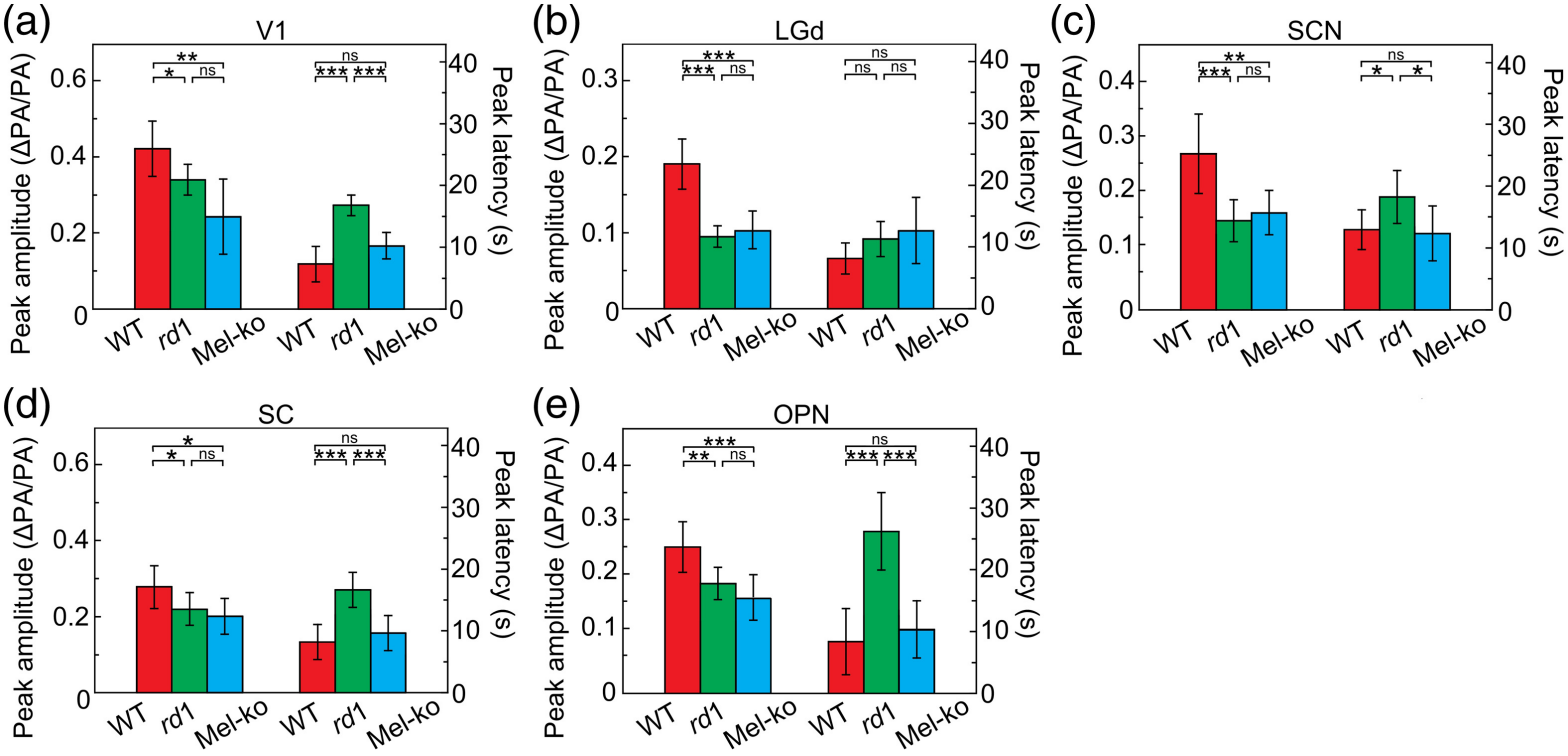
Statistical analysis comparing the peak amplitudes and peak latencies of the targeted nuclei (a)–(e) for wild-type mice (red columns), *rd1* mice (green columns), and mel-KO mice (blue columns). *p<0.05 (n=10,10); **p<0.01 (n=10,10); ***p<0.005 (n=10,10).

Figure 6 shows the statistical analysis of the quantitative measurements from the three genotypes. In all of the targeted nuclei, the peak amplitudes of both *rd1* and mel-KO mice were significantly lower than wild-type mice, but only *rd1* mice had significantly longer response latencies compared with wild-type mice, specifically in V1, SC, OPN, and SCN [Figs. 6(a), 6(b), 6(d), and 6(e)].

## 4 Discussion

Our PACT-US system effectively delineated visual-evoked responses in V1 and several subcortical nuclei in the mouse brain. The quantitative evaluation of temporal traces from these functionally important regions revealed differences in visual-evoked HR among wild-type, *rd1*, and mel-KO mice. These differences are largely congruent with expectations as follows: (1) because all five areas receive input from photoreceptive ganglion cells,^37^^–^^41^ mel-KO mice had lower amplitude photoresponses than wild type; (2) the residual responses in mel-KO were driven by rod/cone photoreceptors and thus were not significantly delayed compared with wild type; and (3) *rd1* mice, using only melanopsin, which responds to light sluggishly,^42^ showed both smaller and slower responses compared with wild type. Such agreement underscores the potential of this imaging technique in high-throughput phenotyping of the mouse brain.

Currently, the frame rate of the imaging system is 3.3 Hz at each imaging plane. The temporal traces in Figs. 4 and 5 show that this frame rate can capture the HR waveform with sufficient fidelity to detect the reduced response kinetics in *rd1* mice. The limiting factors of the current imaging system include the 2D imaging ability, the small number of imaging planes, the mechanical translation between the imaging planes, and the laser repetition rate. The frame rate can be potentially improved using 2D US transducer arrays for 3D imaging without mechanical translation and lasers with higher repetitional rates. A substantial increase in frame rate will allow more temporal averaging to increase the signal-to-noise ratios and the potential detection of more subtle features in HRs such as the overshoot and undershoot observed in fMRI.^43^^,^^44^

The 797-nm optical wavelength was used for PA excitation in this study to target total hemoglobin content. All of the quantitative analysis was based on PA signal changes. The optical attenuation along the penetration was not considered in this study. This approach is comparable to fMRI sensitized to the contrast of cerebral blood volume, which has been shown to have greater spatial specificity of neural activity detection compared with BOLD contrast.^45^ The measurement of oxygenation with PACT would require at least two optical wavelengths, which attenuate differently as they penetrate the brain. Signal intensity compensation using optical energy distribution at each wavelength, as was done in previous studies,^46^^,^^47^ would have to be performed for quantitative analysis.

The temporal traces in Fig. 4 showed changes with ∼40% fluctuations. Considering that there is an ∼10% system fluctuation at the resting state, the visually evoked signal has an ∼30% fractional change, which is comparable to a previous study.^48^ The post-stimulus undershoot has been observed in fMRI and fNIR signals in both humans and animals.^43^ The empirical canonical HR function integrates such features.^25^^–^^27^ We believe that the negative changes of the HR in this study are real and captured by the canonical function fitting. In our future work, we will seek to define the true hemodynamics response function in the visual region using larger cohorts and animals and improve our results. We include this information in the discussion section.

A unique advantage of PACT in small animals, similar to that of fNIRS in human subjects,^49^ is the feasibility of integrating all imaging components into a small and lightweight wearable 3D platform for brain imaging. Such a platform would allow for volumetric imaging of multiple visual nuclei in mice without anesthesia or restraining, thereby avoiding anesthesia/restraint-induced HR alterations as well as facilitating longer-term recordings. Numerous studies have reported that anesthesia inhibits neural responses in the visual system. For instance, we have shown that raising the isoflurane level from 0.5% to 1% dramatically slows pupillary light reflexes in mice.^20^ Other labs have found anesthesia to render SCN photoresponses significantly weaker^50^ and more transient^51^^,^^52^ and to disrupt higher visual processing.^53^^–^^57^ PACT imaging of free-moving animals would enable studying visual responses under more normal physiologic conditions.

## 5 Conclusion

This study has established a label-free, high-resolution PACT system capable of real-time monitoring of visual-evoked HRs across different brain regions in anesthetized mice. The system successfully identified hemodynamic changes in V1, SC, LGd, OPN, and SCN in response to retinal photostimulation. Considering that (1) these are the only five visual regions the we have attempted to date and all were imaged successfully and (2) the SCN is likely one of the most challenging nuclei to image given its depth and small size, it seems reasonable to expect PACT to be capable of imaging most of the other visual areas in the mouse brain. Comparisons among the responses in *rd1*, mel-KO, and wild-type mice validated the sensitivity of this technique in discerning significant variances in response amplitudes and latencies. Such findings highlight the potential of our system to detect post-retinal visual deficits in various mouse models, filling a significant technological gap in vision research.

## Data Availability

Data and data processing codes underlying the results presented in this paper are stored at an online archiving location that may be accessed from the authors upon reasonable request.

## References

[1] Won J. et al., “Mouse model resources for vision research,” J. Ophthalmol. 2011, 391384 (2011). 10.1155/2011/391384 21052544 PMC2968714

[2] Won J. et al., “Translational vision research models program,” in Retinal Degenerative Diseases, LaVail M. et al., Eds., pp. 391–397, Springer US (2012). 10.1007/978-1-4614-0631-0_50 22183357

[3] Wan X. et al., “The neural basis of the hemodynamic response nonlinearity in human primary visual cortex: implications for neurovascular coupling mechanism,” NeuroImage 32, 616–625 (2006). NEIMEF 1053-8119 10.1016/j.neuroimage.2006.03.040 16697664

[4] Niranjan A. et al., “fMRI mapping of the visual system in the mouse brain with interleaved snapshot GE-EPI,” NeuroImage 139, 337–345 (2016). NEIMEF 1053-8119 10.1016/j.neuroimage.2016.06.015 27296012 PMC4988789

[5] Strangman G. et al., “A quantitative comparison of simultaneous BOLD fMRI and NIRS recordings during functional brain activation,” NeuroImage 17, 719–731 (2002). NEIMEF 1053-8119 10.1006/nimg.2002.1227 12377147

[6] Ayaz H. et al., “Optical imaging and spectroscopy for the study of the human brain: status report,” Neurophotonics 9, S24001 (2022). 10.1117/1.NPh.9.S2.S24001 36052058 PMC9424749

[7] Das D. et al., “Label-free high frame rate imaging of circulating blood clots using a dual modal ultrasound and photoacoustic system,” J. Biophotonics 14, e202000371 (2021). 10.1002/jbio.202000371 33231356

[8] Wang L. V. Hu S., “Photoacoustic tomography: in vivo imaging from organelles to organs,” Science 335, 1458–1462 (2012). SCIEAS 0036-8075 10.1126/science.1216210 22442475 PMC3322413

[9] Nasiriavanaki M. et al., “High-resolution photoacoustic tomography of resting-state functional connectivity in the mouse brain,” Proc. Natl. Acad. Sci. 111, 21 (2014). 10.1073/pnas.1311868111 24367107 PMC3890828

[10] Zhang P. et al., “High-resolution deep functional imaging of the whole mouse brain by photoacoustic computed tomography in vivo,” J. Biophotonics 11, e201700024 (2018). 10.1002/jbio.201700024 PMC5777675 28635056

[11] Yao J. et al., “Noninvasive photoacoustic computed tomography of mouse brain metabolism in vivo,” NeuroImage 64, 257–266 (2013). NEIMEF 1053-8119 10.1016/j.neuroimage.2012.08.054 22940116 PMC3508393

[12] Gottschalk S. et al., “Rapid volumetric optoacoustic imaging of neural dynamics across the mouse brain,” Nat. Biomed. Eng. 3, 392–401 (2019). 10.1038/s41551-019-0372-9 30992553 PMC6825512

[13] Chang K.-W. et al., “Label-free photoacoustic computed tomography of mouse cortical responses to retinal photostimulation using a pair-wise correlation map,” Biomed. Opt. Express 13, 1017–1025 (2022). BOEICL 2156-7085 10.1364/BOE.446990 35284169 PMC8884203

[14] Tang J. et al., “Noninvasive high-speed photoacoustic tomography of cerebral hemodynamics in awake-moving rats,” J. Cereb. Blood Flow Metab. 35, 1224–1232 (2015). 10.1038/jcbfm.2015.138 26082016 PMC4527999

[15] Tang J. et al., “Wearable 3-D photoacoustic tomography for functional brain imaging in behaving rats,” Sci. Rep. 6, 25470 (2016). SRCEC3 2045-2322 10.1038/srep25470 27146026 PMC4857106

[16] Chang K.-W. et al., “Photoacoustic imaging of visually evoked cortical and subcortical hemodynamic activity in mouse brain: feasibility study with piezoelectric and capacitive micromachined ultrasonic transducer (CMUT) arrays,” Biomed. Opt. Express 14, 6283–6290 (2023). BOEICL 2156-7085 10.1364/BOE.503475 38420324 PMC10898584

[17] Ecker J. L. et al., “Melanopsin-expressing retinal ganglion-cell photoreceptors: cellular diversity and role in pattern vision,” Neuron 67, 49–60 (2010). NERNET 0896-6273 10.1016/j.neuron.2010.05.023 20624591 PMC2904318

[18] Shih A. Y. et al., “A polished and reinforced thinned-skull window for long-term imaging of the mouse brain,” J. Visualized Exp. 61, e3742 (2012). 10.3791/3742 PMC3460568 22433225

[19] Yang G. et al., “Thinned-skull cranial window technique for long-term imaging of the cortex in live mice,” Nat. Protoc. 5, 201–208 (2010).1754-2189 10.1038/nprot.2009.222 20134419 PMC4690457

[20] Eckley S. S. et al., “Acepromazine and chlorpromazine as pharmaceutical-grade alternatives to chlorprothixene for pupillary light reflex imaging in mice,” J. Amer. Assoc. Lab Anim. Sci. 59, 197–203 (2020). 10.30802/AALAS-JAALAS-19-000094 31915106 PMC7073400

[21] Ma Y. et al., “Mammalian near-infrared image vision through injectable and self-powered retinal nanoantennae,” Cell 177, 243–255.e15 (2019). CELLB5 0092-8674 10.1016/j.cell.2019.01.038 30827682

[22] Luo D. G. et al., “Activation of visual pigments by light and heat,” Science 332, 1307–1312 (2011). SCIEAS 0036-8075 10.1126/science.1200172 21659602 PMC4349410

[23] A. Standard Z136.1, American National Standard for the Safe Use of Lasers, American National Standards Institute, Inc., New York (1993).

[24] Gagnon L. et al., “Improved recovery of the hemodynamic response in diffuse optical imaging using short optode separations and state-space modeling,” NeuroImage 56, 1362–1371 (2011). NEIMEF 1053-8119 10.1016/j.neuroimage.2011.03.001 21385616 PMC3085546

[25] Henson R. Friston K., “Convolution models for fMRI,” in Statistical parametric mapping: the analysis of functional brain images, Friston K. et al., Eds., pp. 178–192, Academic Press (2007).

[r26] Jin T. Kim S.-G., “Cortical layer-dependent dynamic blood oxygenation, cerebral blood flow and cerebral blood volume responses during visual stimulation,” NeuroImage 43, 1–9 (2008). NEIMEF 1053-8119 10.1016/j.neuroimage.2008.06.029 18655837 PMC2579763

[27] Havlicek M. et al., “Determining excitatory and inhibitory neuronal activity from multimodal fMRI data using a generative hemodynamic model,” Front. Neurosci. 11, 616 (2017).1662-453X 10.3389/fnins.2017.00616 29249925 PMC5715391

[28] McKeown M. J. Hansen L. K. Sejnowsk T. J., “Independent component analysis of functional MRI: what is signal and what is noise?” Curr. Opin. Neurobiol. 13, 620–629 (2003). COPUEN 0959-4388 10.1016/j.conb.2003.09.012 14630228 PMC2925426

[29] Hernandez-Martin E. et al., “Diffuse optical tomography to measure functional changes during motor tasks: a motor imagery study,” Biomed. Opt. Express 11, 6049–6067 (2020). BOEICL 2156-7085 10.1364/BOE.399907 33282474 PMC7687968

[30] Cai Z. et al., “Diffuse optical reconstructions of functional near infrared spectroscopy data using maximum entropy on the mean,” Sci. Rep. 12, 2316 (2022). SRCEC3 2045-2322 10.1038/s41598-022-06082-1 35145148 PMC8831678

[31] Han K. et al., “Variational autoencoder: an unsupervised model for encoding and decoding fMRI activity in visual cortex,” NeuroImage 198, 125–136 (2019). NEIMEF 1053-8119 10.1016/j.neuroimage.2019.05.039 31103784 PMC6592726

[r32] Cignetti F. et al., “Pros and cons of using the informed basis set to account for hemodynamic response variability with developmental data,” Front. Neurosci. 10, 322 (2016).1662-453X 10.3389/fnins.2016.00322 27471441 PMC4945642

[r33] Kim D. H. et al., “Increasing motor cortex activation during grasping via novel robotic mirror hand therapy: a pilot fNIRS study,” J. NeuroEng. Rehabil. 19, 8 (2022). 10.1186/s12984-022-00988-7 35073933 PMC8785601

[r34] Calhoun V. D. et al., “fMRI analysis with the general linear model: removal of latency-induced amplitude bias by incorporation of hemodynamic derivative terms,” NeuroImage 22, 252–257 (2004). NEIMEF 1053-8119 10.1016/j.neuroimage.2003.12.029 15110015

[r35] Lambers H. et al., “A cortical rat hemodynamic response function for improved detection of BOLD activation under common experimental conditions,” NeuroImage 208, 116446 (2020). NEIMEF 1053-8119 10.1016/j.neuroimage.2019.116446 31846759

[36] Lindquist M. A. et al., “Modeling the hemodynamic response function in fMRI: efficiency, bias and mis-modeling,” NeuroImage 45, S187–S198 (2009). NEIMEF 1053-8119 10.1016/j.neuroimage.2008.10.065 19084070 PMC3318970

[37] Güler A. D. et al., “Melanopsin cells are the principal conduits for rod-cone input to non-image-forming vision,” Nature 453, 102–105 (2008). 10.1038/nature06829 18432195 PMC2871301

[r38] Göz D. et al., “Targeted destruction of photosensitive retinal ganglion cells with a saporin conjugate alters the effects of light on mouse circadian rhythms,” PLoS One 3, e3153 (2008). POLNCL 1932-6203 10.1371/journal.pone.0003153 18773079 PMC2519834

[r39] Hatori M. et al., “Inducible ablation of melanopsin-expressing retinal ganglion cells reveals their central role in non-image forming visual responses,” PLoS One 3, e2451 (2008). POLNCL 1932-6203 10.1371/journal.pone.0002451 18545654 PMC2396502

[r40] Zhao X. et al., “Photoresponse diversity among the five types of intrinsically photosensitive retinal ganglion cells,” J. Physiol. 592, 1619–1636 (2014). JPHYA7 0022-3751 10.1113/jphysiol.2013.262782 24396062 PMC3979615

[41] Brown T. M. et al., “Melanopsin contributions to irradiance coding in the thalamo-cortical visual system,” PLoS Biol. 8, e1000558 (2010). 10.1371/journal.pbio.1000558 21151887 PMC2998442

[42] Berson D. M. Dunn F. A. Takao M., “Phototransduction by retinal ganglion cells that set the circadian clock,” Science 295, 1070–1073 (2002). SCIEAS 0036-8075 10.1126/science.1067262 11834835

[43] Schroeter M. L. et al., “Investigating the post-stimulus undershoot of the BOLD signal—a simultaneous fMRI and fNIRS study,” NeuroImage 30, 349–358 (2006). NEIMEF 1053-8119 10.1016/j.neuroimage.2005.09.048 16257236

[44] van Zijl P. C. M. Hua J. Lu H., “The BOLD post-stimulus undershoot, one of the most debated issues in fMRI,” NeuroImage 62, 1092–1102 (2012). NEIMEF 1053-8119 10.1016/j.neuroimage.2012.01.029 22248572 PMC3356682

[45] Zhao F. et al., “Spatial specificity of cerebral blood volume-weighted fMRI responses at columnar resolution,” NeuroImage 27, 416–424 (2005). NEIMEF 1053-8119 10.1016/j.neuroimage.2005.04.011 15923128

[46] Cox B. T. et al., “Two-dimensional quantitative photoacoustic image reconstruction of absorption distributions in scattering media by use of a simple iterative method,” Appl. Opt. 45, 1866–1875 (2006). APOPAI 0003-6935 10.1364/AO.45.001866 16572706

[47] Cox B. et al., “Quantitative spectroscopic photoacoustic imaging: a review,” J. Biomed. Opt. 17, 061202 (2012). JBOPFO 1083-3668 10.1117/1.JBO.17.6.061202 22734732

[48] Yao J. et al., “High-speed label-free functional photoacoustic microscopy of mouse brain in action,” Nat. Methods 12, 407–410 (2015).1548-7091 10.1038/nmeth.3336 25822799 PMC4428901

[49] von Lühmann A. et al., “Toward Neuroscience of the Everyday World (NEW) using functional near-infrared spectroscopy,” Curr. Opin. Biomed. Eng. 18, 100272 (2021). 10.1016/j.cobme.2021.100272 33709044 PMC7943029

[50] Meijer J. H. et al., “Light responsiveness of the suprachiasmatic nucleus: long-term multiunit and single-unit recordings in freely moving rats,” J. Neurosci. 18, 9078–9087 (1998). JNRSDS 0270-6474 10.1523/JNEUROSCI.18-21-09078.1998 9787011 PMC6793532

[51] van Diepen H. C. et al., “Irradiance encoding in the suprachiasmatic nuclei by rod and cone photoreceptors,” FASEB J. 27, 4204–4212 (2013). FAJOEC 0892-6638 10.1096/fj.13-233098 23796782

[52] Drouyer E. et al., “Responses of suprachiasmatic nucleus neurons to light and dark adaptation: relative contributions of melanopsin and rod–cone inputs,” J. Neurosci. 27, 9623–9631 (2007). JNRSDS 0270-6474 10.1523/JNEUROSCI.1391-07.2007 17804622 PMC6672968

[53] Ghita A. M. et al., “Analysis of the visual evoked potential in anesthesia with sevoflurane and chloral hydrate: (variability of amplitudes, latencies and morphology of VEP with the depth of anesthesia),” J. Med. Life 6, 214–225 (2013). 23904886 PMC3725452

[r54] Nassi J. J. Lomber S. G. Born R. T., “Corticocortical feedback contributes to surround suppression in V1 of the alert primate,” J. Neurosci. 33, 8504–8517 (2013). JNRSDS 0270-6474 10.1523/JNEUROSCI.5124-12.2013 23658187 PMC3690087

[r55] Briggs F. Usrey W. M., “Corticogeniculate feedback and visual processing in the primate,” J. Physiol. 589, 33–40 (2011). JPHYA7 0022-3751 10.1113/jphysiol.2010.193599 20724361 PMC3039257

[r56] Lee H. et al., “Differential effect of anesthesia on visual cortex neurons with diverse population coupling,” Neuroscience 458, 108–119 (2021). 10.1016/j.neuroscience.2020.11.043 33309966 PMC7925367

[57] Lamme V. A. F. Zipser K. Spekreijse H., “Figure-ground activity in primary visual cortex is suppressed by anesthesia,” Proc. Natl. Acad. Sci. 95, 3263–3268 (1998). 10.1073/pnas.95.6.3263 9501251 PMC19730

